# Equivalent benefit of mTORC1 blockade and combined PI3K-mTOR blockade in a mouse model of tuberous sclerosis

**DOI:** 10.1186/1476-4598-8-38

**Published:** 2009-06-15

**Authors:** Kristen Pollizzi, Izabela Malinowska-Kolodziej, Michael Stumm, Heidi Lane, David Kwiatkowski

**Affiliations:** 1 Translational Medicine Division, Department of Medicine, Brigham and Women's Hospital, Boston, Massachusetts 02115, USA; 2 Novartis Institutes For BioMedical Research, Oncology Basel, Novartis Pharma AG, Switzerland

## Abstract

**Background:**

Tuberous sclerosis (TSC) is a hamartoma syndrome in which renal and lung tumors cause the greatest morbidity. Loss of either TSC1 or TSC2 in TSC hamartomas leads to activation of mTORC1 and suppression of AKT. Recent studies indicate that inhibition of mTORC1 with RAD001 (everolimus) leads to rebound activation of AKT, which could protect tumors from drug-induced cell death. Here we examine the potential benefit of inhibition of both mTOR and AKT signaling in a mouse model of TSC, using a dual pan class I PI3K/mTOR catalytic small molecule inhibitor NVP-BEZ235.

**Results:**

Using ENU to enhance *Tsc2*^+- ^kidney tumor development, both RAD001 (10 mg/kg PO 5 d/week) and NVP-BEZ235 (45 mg/kg PO QD) had equivalent effects in suppressing tumor development during a 4 week treatment period, with a 99% reduction in tumor cell mass. Marked reduction in activation of mTORC1, induction of cell cycle arrest, and absence of apoptotic cell death was seen in mice treated with either drug. However, when either was discontinued, there was prompt recovery of tumor growth, with extensive proliferation.

**Conclusion:**

Both mTORC1 blockade alone and combined PI3K-mTOR blockade lead to suppression of tumor development but not tumor elimination in this TSC model.

## Background

Tuberous sclerosis (TSC) is an autosomal dominant tumor suppressor gene syndrome, in which involvement of the brain, kidneys, and lungs cause the greatest clinical problems [1]. Seizures, mental retardation, developmental delay, and autistic features are common during childhood in these patients, and in many patients these clinical issues persist into adulthood. However, after puberty, additional major clinical problems in TSC are the progressive development of renal angiomyolipoma (AML) and pulmonary lymphangioleiomyomatosis (LAM) [2,3]. Both of these lesions are made up of unusual smooth muscle-like and other cells which express both melanosomal markers and VEGF-D. Although progression of these lesions is quite variable from patient to patient, loss of renal function due to either progressive growth of AMLs and/or conversion into a malignant renal tumor is seen in about 5% of TSC patients. Pulmonary LAM is seen nearly exclusively in female TSC patients, and leads to progressive respiratory limitation and failure also in about 5%. LAM is also seen independent of the TSC syndrome, typically in a more severe and progressive form.

Tsc mouse models have been generated consisting of both knock out and conditional alleles of *Tsc1 *and *Tsc2 *[4,5]. None of these replicates the typical features of AML/LAM, though renal epithelial tumors are a consistent development in both *Tsc1*^+- ^and *Tsc2*^+- ^mice, which progress over a period of many months from pure cysts to papillary adenomas to renal carcinoma.

The TSC1 and TSC2 proteins form a tight complex, which functions in a conserved signaling pathway to regulate the kinase activity of mTORC1, through regulation of the state of GTP loading of Rheb [6,7]. Several studies have shown the benefit of rapamycin and other mTORC1 inhibitors in the treatment and prevention of renal tumors in Tsc mouse models [8,9].

Loss of TSC1/TSC2 in TSC hamartomas leads to both activation of mTORC1, as well as feedback inhibition of AKT, through downregulation of IRS and PDGFR expression and other mechanisms [10,11]. This has led to speculation that treatment of TSC hamartomas with mTORC1 inhibitors might lead to restoration of AKT activation, as seen in vitro with treatment of TSC1/TSC2 null cells, and in some patients with malignant disease [12], which may compromise clinical benefit. Here, we explore the potential benefit of the mTORC1 inhibitor RAD001 (everolimus) in comparison to a dual pan-class I PI3K/mTOR catalytic inhibitor NVP-BEZ-235 [13] in the therapy of Tsc2 mouse kidney tumors.

## Methods

### Mouse procedures

*Tsc2*^+- ^mice, originally generated in this laboratory [4], were serially crossed with C57BL/6J mice for over 5 generations, and were then mated with pure 129S1/SvImJ mice to generate *Tsc2*^+- ^mixed strain C57BL/6J:129S1/SvImJ mice. These mixed strain mice were used in all experiments. All procedures were carried out in accordance with the Guide for the Humane Use and Care of Laboratory Animals, and the study was approved by the Animal Care and Use Committee of Children's Hospital, Boston. N-ethyl-N-nitrosourea (ENU, Sigma-Aldrich) was prepared in ethanol at 200 mg/ml, diluted in phosphate-citrate buffer, and administered by intraperitoneal (IP) injection at 60 mg/kg.

### Standard histology and tumor assessment

Standard histology sections were prepared from mouse kidneys after 10% formalin fixation and cutting into five 1–2 mm sections. Both gross and microscopic kidney pathology was read by a blinded observer (KP) and scored according to a modification of a formula used previously [14]. The kidney tumor score for kidney cystadenomas was determined as a summed score for all lesions in a kidney, scoring each individual tumor grossly as follows: 1 for tumors <1 mm; 2 for 1 to 1.5 mm; 5 for 1.5 to 2 mm; 10 for > 2 mm. Microscopic kidney tumor scores were determined similarly, except that the score for each lesion was multiplied by 2 if the tumor had a papillary component, and by 4 if it was a solid adenoma. The percent cellularity of cystadenomas was determined as the percent of the tumor that contained proliferating cells as opposed to cyst cavity; pure cysts had a score of 0% cellularity while solid adenomas had a score of 100% cellularity. Comparison between sets of mice for tumor measurements were made using the non-parametric Kruskal-Wallis test.

### Antibodies and immunohistochemistry

Antibodies used were: pS6(S235/236) clone 91B2, active caspase-3 clone 5A1 from Cell Signaling Technology, Bedford, MA; pS6(S240) clone DAK-S6-240 M7300, pAKT(S473) clone 14–5, Ki-67 clone TEC-3 M7249, and pMAPK(pTpY202/4) clone 24-2-2 from DAKO S/A, Denmark; PCNA (PC10) from Santa Cruz Biotechnology. For immunohistochemistry, kidneys were rapidly removed, sliced in thirds and fixed overnight at 4°C in 10% formalin. Paraffin sections were cut and stained by the immunoperoxidase technique, following standard methods of deparaffinization, antigen retrieval using Dako Target Retrieval solution, DAB incubation, and counterstaining with hematoxylin. The percent Ki-67 or PCNA labeling, or percent of cells expressing active caspase 3, within a lesion was determined by direct counting of at least 300 cells by a blinded observer (IM).

### Drug handling and administration

RAD001 was provided by Novartis in a proprietary vehicle at 20 mg/ml. Prior to each administration, RAD001 was diluted in water to 0.5–2 mg/ml, and was given at 10 mg/kg by gavage every day 5 days per week. NVP-BEZ235 was provided by Novartis as a powder, and was mixed in 10% 1-Methyl-2-pyrrolidone and 90% PEG-300 at 8 mg/ml. It was prepared fresh prior to each administration, and was given at either 15 mg/kg or 45 mg/kg by gavage every day. In the first treatment cohort, mice were treated with placebo (10% 1-Methyl-2-pyrrolidone and 90% PEG-300) by gavage 5 days per week. This was not done in subsequent cohorts.

## Results

### ENU acts as a carcinogen to enhance renal tumor growth in *Tsc2*^+- ^mice

Kidney tumor growth in *Tsc2*^+- ^mice follows a variable though predictable pattern with an effect of strain on tumor severity [4], (unpublished). In most strains, age 12 months is the earliest at which significant kidney tumors are seen. To accelerate the rate of development of renal tumors, we treated *Tsc2*^+- ^mice with ENU, an alkylating agent which causes point mutations. We explored the effects of administration of a single IP dose of ENU at several different times during mouse development, evaluating tumor severity at 6 months of age [see Additional file 1]. To minimize effects of genetic background, we studied mice that were heterozygous for the C57BL/6J and 129S1/SvImJ strains. We found that ENU given at ages from E13 to P21 (administered to the pregnant dam in the first case) was effective at increasing the incidence and severity of kidney cystadenomas in the *Tsc2*^+- ^mice, as assessed by gross evaluation [see Additional file 1]. ENU treatment at these ages in wild type mice of the same strain mix led to rare renal tumors (e.g. only one < 1 mm tumor in 10 mice treated with ENU at P9) at age 6 months. Microscopic assessment of the ENU-treated *Tsc2*^+- ^mice confirmed this enhancement in tumor development (data not shown). Administration of ENU at any of P9, E19, or E13 appeared to have similar outcomes, with nearly identical kidney tumor scores (see Methods for details) [see Additional file 1]. We chose to use P9 administration for simplicity in the following studies.

### Evaluation of RAD001 alone and in combination with NVP-BEZ235 in the ENU-accelerated *Tsc2*^+- ^kidney tumor model

**Figure 1:**
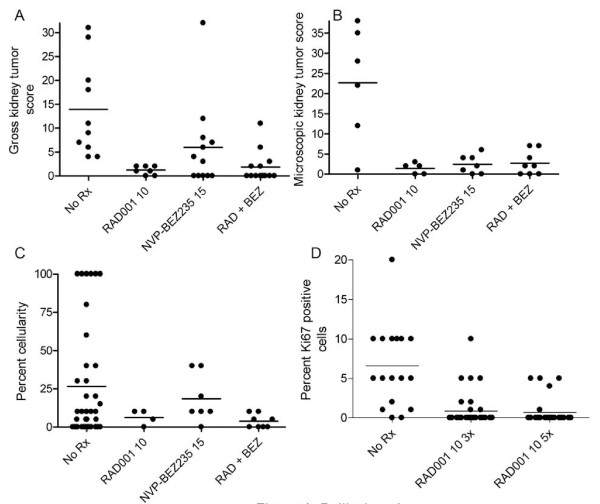
**Renal tumor development blocked by administration of RAD001 with or without NVP-BEZ235 in the ENU-treated *Tsc2*^+- ^model**. A-C. Dot plot analysis of gross kidney tumor score (A), microscopic kidney tumor score (B), and percent cellularity of each lesion (C) in ENU-treated *Tsc2*^+- ^mice at age 24 weeks that received either 1) placebo, 2) RAD001 10 mg/kg PO 5 d/week, 3) NVP-BEZ235 15 mg/kg PO QD, or 4) a combination of RAD001 and NVP-BEZ235 at the same dosage; all for four weeks from age 20 – 24 weeks. A, B: Each dot represents a mouse kidney. C: Each dot represents a mouse kidney cystadenoma. A, p < .001; B, p = .03; C, p NS; by Kruskal-Wallis test. D. Dot plot analysis of percent Ki-67+ cells in kidney tumors of mice with genotype and treatment as in A-C, but that were analyzed 3 or 5 days after initiation of treatment with RAD001 10 mg/kg PO QD at age 24 weeks. Each dot represents a mouse kidney cystadenoma. p < .0001 Kruskal-Wallis test.

We explored the potential benefit of mTORC1 inhibition with RAD001 in the ENU-accelerated *Tsc2*^+- ^kidney tumor model. RAD001 was highly effective in reducing the gross tumor score, microscopic tumor score, and percent solid tumor (Figure 1A–C) in these mice, after a 4-week period of treatment at 10 mg PO QD 5 days out of seven each week, beginning at age 20 weeks. Combining the reduction in overall tumor size with reduction in cellularity indicates that there was an approximate 99% reduction in tumor development. In addition, the residual lesions seen in the RAD001-treated mice generally had a flattened epithelium, in contrast to the enlarged columnar-like epithelial cells seen in untreated mice [see Additional file 2]. We also examined the acute effects of treatment with RAD001 in this model. Tumor analysis 3–5 days after initiation of therapy demonstrated that RAD001 markedly reduced expression of pS6(S240) and pS6(S235/236), consistent with mTORC1 blockade [see Additional file 3]. In addition, the Ki-67 labeling index in the short-term treated tumors was reduced from an average of 6% to an average of 1% (Figure 1D) [see Additional file 3]. However, there was no indication of induction of apoptosis or necrosis in the tumors, as expression of activated caspase-3 was very low in the treated tumors (0–1% cells) similar to that seen in untreated tumors [see Additional file 4A] (in contrast to control nude mouse tumor xenograft samples, data not shown). In addition, there was no consistent effect on MAPK signaling, as assessed by staining for pMAPK(pTpY202/4) (data not shown), in the treated tumors. However, RAD001 treatment caused an increase in pAKT(S473) levels in the tumors, which were very low in tumors from untreated mice [see Additional file 5]. Total S6 and AKT protein levels were similar in normal kidney and in the tumors, and did not appear to change significantly with treatment with either compound [see Additional file 5]. Four week treatment with RAD001 also did not lead to significant apoptosis in these kidney tumors, though it did cause continued suppression of proliferation [see Additional file 4].

Simultaneously, we evaluated the potential benefit of combining RAD001 inhibition of mTORC1 with PI3K-mTOR inhibition using NVP-BEZ235. NVP-BEZ235 was also given alone (without RAD001), as a control, and at relatively low dosage (15 mg/kg PO QD) appeared to have significant therapeutic effect in the ENU-treated *Tsc2*^+- ^mice (Figure 1A–C). Improvement was seen in both gross and microscopic kidney tumor scores, with a more modest change in tumor cellularity. These observations are probably due to the activity of NVP-BEZ235 as a direct mTOR inhibitor, affecting both mTORC1 and mTORC2, in addition to its PI3K inhibition activity [13]. Consistent with this effect, NVP-BEZ235 inhibits phosphorylation of S6 at the S235/236 sites in Tsc2 null murine embryo fibroblast cell lines at 10–100 nM, and has a potent anti-proliferative effect on these cells with an IC50 of 3 nM [15].

### Comparison of RAD001 and NVP-BEZ235 as therapy for the ENU-accelerated *Tsc2*^+- ^kidney tumor model

**Figure 2:**
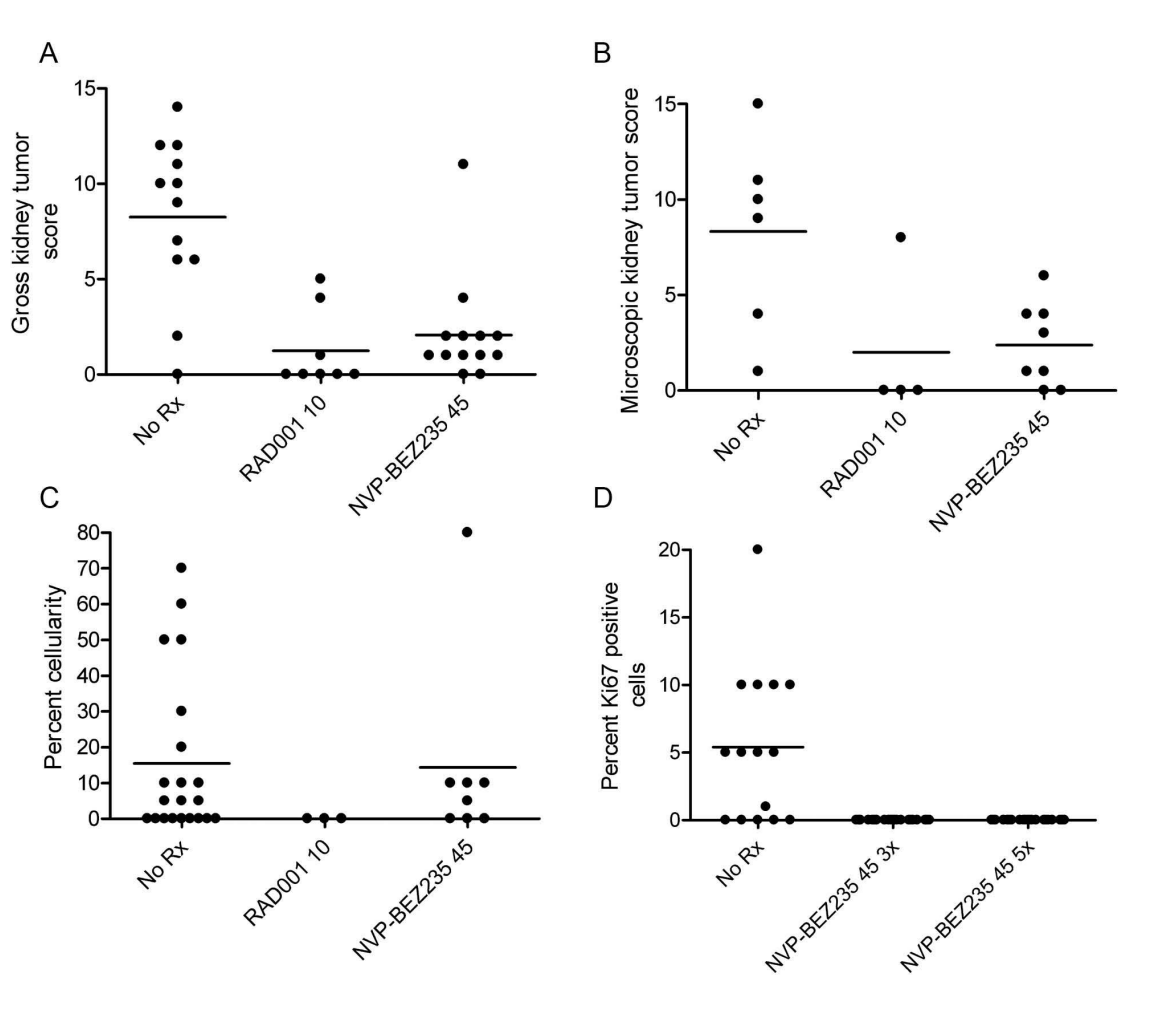
**Renal tumor development blocked by administration of either RAD001 or NVP-BEZ235 in the ENU-treated *Tsc2*^+- ^model**. A-C. Dot plot analysis of gross kidney tumor score (A), microscopic kidney tumor score (B), and percent cellularity of each lesion (C) in ENU-treated *Tsc2*^+- ^mice at age 24 weeks that received either 1) no treatment, 2) RAD001 10 mg/kg PO 5 d/week, or 3) NVP-BEZ235 45 mg/kg PO QD; all for four weeks from age 20 – 24 weeks. A, B: Each dot represents a mouse kidney. C: Each dot represents a mouse kidney cystadenoma. A, p < .001; B, p = .03; C, p NS; by Kruskal-Wallis test. D. Dot plot analysis of percent Ki-67+ cells in kidney tumors of mice with genotype and treatment as in A-C, but that were analyzed 3 or 5 days after initiation of treatment with NVP-BEZ235 45 mg/kg PO QD at age 24 weeks. Each dot represents a mouse kidney cystadenoma. p < .0001 Kruskal-Wallis test.

Since NVP-BEZ235 had effects in inhibiting mTOR, and at low doses could reduce tumor development in this model, we treated a cohort of ENU-treated *Tsc2*^+- ^mice with NVP-BEZ235 at full dosage, 45 mg/kg PO QD [13], and compared outcome with RAD001 treatment. NVP-BEZ235 had similar effects to RAD001 in reducing both gross and microscopic kidney tumor scores by about 80%, with most residual lesions being simple cysts (Figure 2A–C). Tumor cellularity also appeared reduced in general, though the presence of a single solid adenoma in an NVP-BEZ235-treated mouse ran against this trend. Tumor cell size was reduced in NVP-BEZ235-treated mice [see Additional file 2]. In short term as well as 4 week treatment trials, NVP-BEZ235 stopped cell proliferation, with complete loss of Ki-67 or PCNA staining in the treated tumors (Figure 2D) [see Additional files 3, 4]. Similar to RAD001, NVP-BEZ235 did not appear to cause apoptosis in the tumor cells [see Additional file 4A]. pS6(S240) and pS6(S235/236) expression was markedly reduced in the 5 day NVP-BEZ235-treated mouse kidney tumors [see Additional file 3]. pAKT(S473) levels were low in the NVP-BEZ235-treated mouse tumors, similar to untreated mice, but in contrast to RAD001-treated mice [see Additional file 5]. There was no statistically significant difference between the kidney tumor scores or cellularity of the tumors seen in these mice after RAD001 or NVP-BEZ235 treatment.

### Comparison of RAD001 and NVP-BEZ235 as therapy for the ENU-accelerated *Tsc2*^+- ^kidney tumor model with long-term follow-up

**Figure 3:**
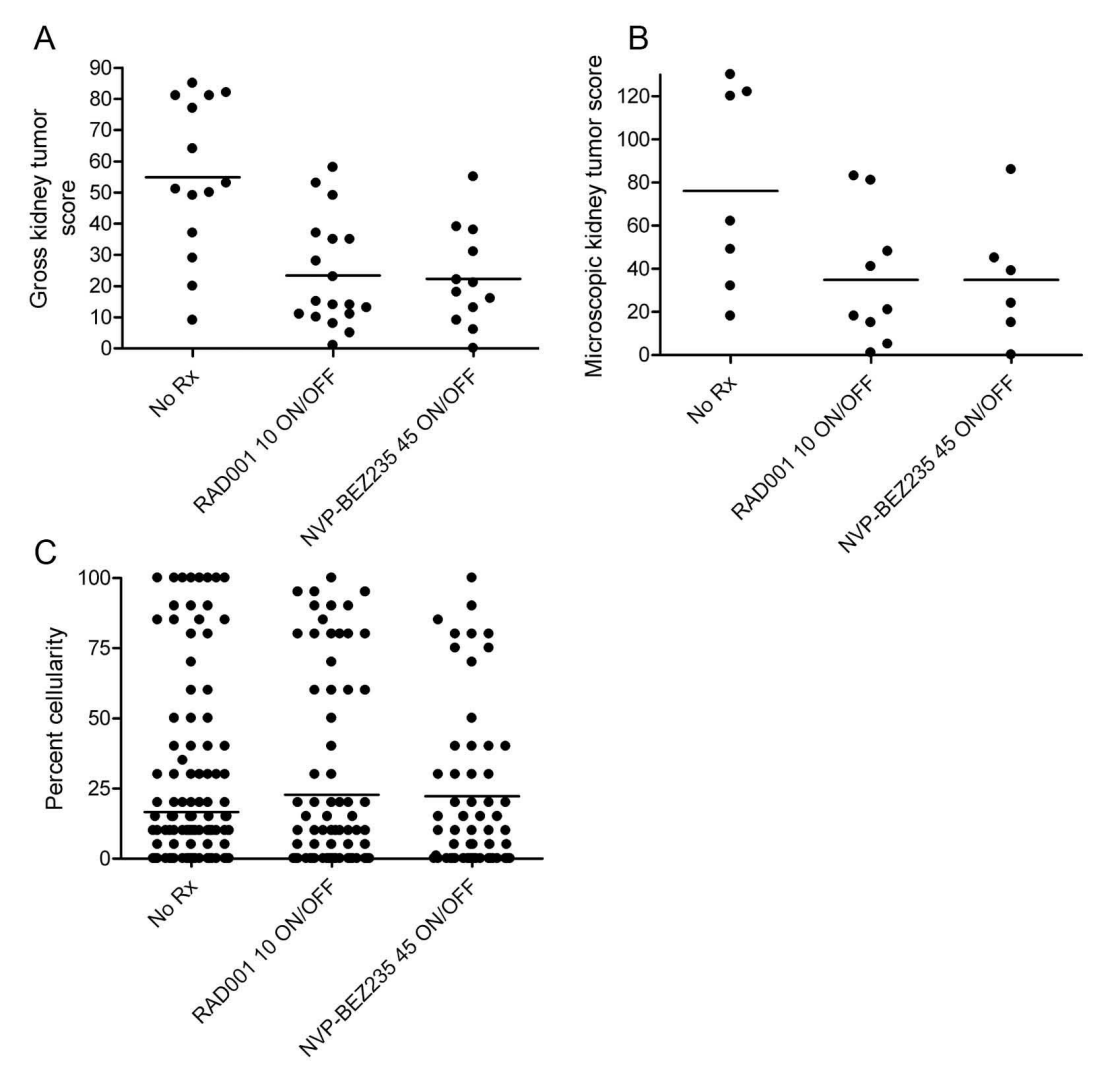
**Renal tumor recurrence in mice treated with either RAD001 or NVP-BEZ235 8 weeks after coming off drug**. A-C. Dot plot analysis of gross kidney tumor score (A), microscopic kidney tumor score (B), and percent cellularity of each lesion (C) in ENU-treated *Tsc2*^+- ^mice at age 32 weeks that received either 1) no treatment, 2) RAD001 10 mg/kg PO 5 d/week, or 3) NVP-BEZ235 45 mg/kg PO QD; all for four weeks from age 20 – 24 weeks. A, B: Each dot represents a mouse kidney. C: Each dot represents a mouse kidney cystadenoma. A, p = .002; B and C, p NS; by Kruskal-Wallis test.

Since RAD001 and NVP-BEZ235 had similar effects in arresting the growth of the kidney tumor epithelial cells during a four week period of treatment, we asked whether one or the other treatment might be more beneficial in terms of lasting effects on tumor growth in mice treated transiently. To explore this question, mice were treated with either drug for a period of 4 weeks, age 20 – 24 weeks, and then were taken off drug for 8 weeks and sacrificed for examination. Kidney tumors in mice treated with either drug showed robust growth with development of relatively large papillary and solid tumors, and re-expression of PCNA [see Additional file 4B]. Gross tumor scores were significantly reduced in mice treated with either drug in comparison to never-treated mice; however, there was no significant difference in microscopic tumor scores or percent cellularity (Figure 3) [see Additional file 2]. Gross and microscopic kidney tumor scores, percent papillary and solid tumors, and general histologic characteristics of the tumors in these mice did not differ according to the drug treatment received. Thus, both RAD001 and NVP-BEZ235 had major effects on tumor growth during the treatment period, but resumption of brisk tumor growth occurred upon cessation of treatment.

## Discussion

Tuberous sclerosis affects an estimated 40,000 individuals in the United States, and about 10% of these individuals are projected to sustain significant morbidity and often mortality due to development and progression of renal AML, pulmonary LAM, other less common tumors of the retroperitoneum (lymphangioma) or liver (angiomyolipoma), PEComas arising in various sites, and neuroendocrine tumors [1]. The major understanding of the function of the TSC1/TSC2 protein complex as a critical regulator of the Rheb GTPase and thereby mTORC1 activity in recent years has led to considerable enthusiasm about the potential efficiency of rapamycin and other mTORC1 inhibitors as therapy for these TSC tumors [7]. Two phase I-II trials of rapamycin therapy for TSC renal AML and/or pulmonary LAM have been published [16,17]. The larger trial reported that among the 80% patients staying on drug, those with renal AML responded to rapamycin with an average 47% reduction in tumor volume. Although this was gratifying, the reduction in size was largely reversed, returning to 86% of starting volume on average, when follow-up for a year off drug was performed. Since no biopsies were performed in these patients, there is a lack of understanding of precisely what happened to these tumors both when the patients were treated and when the drug was stopped.

Here we demonstrate, similar to previous reports using rapamycin [8,9], that RAD001 is highly effective in suppressing the growth of Tsc mouse kidney cystadenomas, with an average 99% reduction in tumor cell burden in this ENU-accelerated *Tsc2*^+- ^model. In addition, we demonstrate that there is very effective suppression of cell growth within these lesions, as assessed by reduction in Ki-67 positivity, with a lack of apoptosis or tumor cell death. Pathway inhibition with reduction in markers of mTORC1 activation, pS6(S240) and pS6(S235/236), was also seen, consistent with the expected mechanism of action of the drug. However, marked tumor regrowth occurred by 8 weeks after discontinuation of RAD001, though some persistent reduction in overall tumor burden could be appreciated in comparison to never-treated mice (Figure 3A). We find these observations to be in striking parallel to those made in AML patients, taking into account the markedly different growth rates of renal AMLs in patients in comparison to renal cystadenomas in this model. They suggest that rapamycin/RAD001 has a cytostatic effect, effectively blocking the growth of the renal AMLs and likely reducing cell size, but without tumor cell death. Upon drug removal, there is regrowth of tumors. Further, they suggest the possibility that continued mTORC1 inhibition may have benefit for maintaining growth suppression of these tumors.

It is well-recognized that the mTOR signaling pathway in which TSC1/TSC2 participate is more complex than first thought, with a number of feedback inhibitory effects of loss of the TSC protein complex on AKT activation [10,11,18]. In addition, the related mTORC2 complex has a major if not sole role in phosphorylating and enhancing activation of AKT at Serine 473 [19]. In most tumor cell lines, including Tsc1 null and Tsc2 null MEF cell lines, treatment with rapamycin or RAD001 leads to phosphorylation of AKT at the S473 site, and increased activation [11,12,20]. There has been concern that this effect could mitigate any positive treatment effect of mTORC1 inhibitors on tumor growth. Here we assessed the possibility that combined PI3K-AKT-mTOR blockade with NVP-BEZ235 could lead to a better therapeutic outcome in this *Tsc2*^+- ^kidney tumor model. We found that the short-term effects of NVP-BEZ235 were similar to those of RAD001 with a major reduction in cell proliferation, lack of apoptosis or cell death, and reduction in markers of mTORC1 activation. NVP-BEZ235 has PI3K inhibitory activity at low nM levels in vitro for all PI3Kα including mutant forms, and has been shown to reduce pAKT(S473) levels in xenograft models [13]. As expected, pAKT(S473) levels were low in the kidney tumors from untreated *Tsc2*^+- ^mice, and were increased by treatment with RAD001, but not NVP-BEZ235. In addition, in vitro studies demonstrate that in full serum pAKT(S473) levels are low in Tsc2 null MEF lines, are increased somewhat with RAD001 treatment, and reduced somewhat by NVP-BEZ235 treatment [15]. Despite the short-term effects of treatment with NVP-BEZ235, we found that in both the 4 week course of drug, and 4-week course with 8-week off drug follow-up, that RAD001 and NVP-BEZ235 had indistinguishable effects, with marked regrowth of tumor following treatment cessation. Thus, these observations suggest that the reactivation of mTORC1 in TSC-related neoplasms that might occur with rapamycin/RAD001 treatment has no significant clinical effect, at least in this Tsc model tumor.

## Conclusion

We have conducted a trial of the pure mTORC1 inhibitor RAD001 and the combined PI3K/mTOR inhibitor NVP-BEZ235 in a mouse model of TSC in which the mice develop renal cystadenomas. Both drugs were highly effective at tumor growth suppression, and there was no difference between combined PI3K-mTOR blockade in comparison to mTORC1 inhibition alone. When treatment was discontinued, rapid tumor regrowth was seen after each drug. In this model, both drugs appear to have a primarily cytostatic effect.

## Abbreviations

(TSC): Tuberous sclerosis; (AML): angiomyolipoma; (LAM): lymphangioleiomyomatosis; (ENU): N-ethyl-N-nitrosourea.

## Competing interests

This work was partially supported by Novartis Pharma AG.

## Authors' contributions

KP performed most of the experimentation in this project, including mouse treatment, pathology preparation, and tumor scoring. MS and IM performed the immunohistochemical studies and their interpretation. HL assisted in the conception of the study and monitoring during its execution. DJK conceived of the study, monitored the mice and treatment, and drafted the manuscript. All authors contributed to the editing of the manuscript, and approved the final version.

## Supplementary Material

Additional file 1 **Effects of ENU on kidney tumor development in *Tsc2*^+- ^mice**. A dot plot graph is shown of the gross kidney tumor scores in *Tsc2*^+- ^mice at 6 months of age. E13, E19, P9, P21 indicates different ages of treatment with a single dose of ENU at 60 mg/kg IP. Each dot represents a single mouse. The differences among the 5 groups are statistically significant at p = 0.02; for the four ENU treatment groups, p = 0.035; for the three ENU treatment groups of E13, E19, and P9, p is not significant (0.79); all done by the Kruskall-Wallis test. Click here for file

Additional file 2 **Morphology of RAD001- and NVP-BEZ235-treated *Tsc2*^+- ^kidney lesions**. Multiple tumor H&E stained images are shown from ENU-treated *Tsc2*^+- ^kidneys. All scale bars are 100 microns. Each image is taken from a different mouse. Light black lines delineate tumor (T) or cyst (C) vs. normal kidney (N). Row A- Mice of age 24 weeks; inset shows hyperplastic columnar-type epithelium lining the cyst; Row B- Mice of age 24 weeks that were treated with RAD001 5 days per week, from age 20–24 weeks; inset shows flat epithelium lining the cyst: Row C- Mice of age 24 weeks that were treated with NVP-BEZ235 45 mg/kg daily from age 20–24 weeks; Row D- Mice of age 32 weeks; Row E- Mice of age 32 weeks that were treated with RAD001 5 days per week, from age 20–24 weeks; Row F- Mice of age 32 weeks that were treated with NVP-BEZ235 45 mg/kg daily from age 20–24 weeks. Click here for file

Additional file 3 **Signaling pathway and growth analysis by IHC in *Tsc2*^+- ^mice**. A single representative image is shown for untreated mice (top row), mice treated for 5 days with RAD001 10 mg/kg PO (middle row), and mice treated for 5 days with NVP-BEZ235 45 mg/kg PO (bottom row). All mice were ENU-treated *Tsc2*^+- ^mice of age 24 weeks. Columns are: pS6(S240), pS6(S235/236), Ki-67. Adjacent sections from each tumor are shown. All images are taken at 100×. Note that the third row has a background pink/orange shade, which is present in all cells on this section, and distinct from the dark brown stain seen in the top row. Click here for file

Additional file 4 **Apoptosis and proliferation in *Tsc2*^+- ^mice**. A. The percent cleaved caspase 3 positive cells are shown for *Tsc2*^+- ^mice, treated for either 5 days ('SHORT') or for 4 weeks ('CHRONIC'). Each dot represents a different renal cystadenoma for which the percent positive cells was determined by a blinded observer (IM). B. The percent PCNA positive cells are shown for *Tsc2*^+- ^mice, treated with either RAD001 or NVP-BEZ235, or treated with these drugs and then taken off drug for 8 weeks ('ON/OFF'). Each dot represents a different renal cystadenoma. Click here for file

Additional file 5 **Signaling pathway analysis by IHC in *Tsc2*^+-^mice**. A single representative image is shown for untreated mice (top row), mice treated for 5 days with RAD001 10 mg/kg PO (middle row), and mice treated for 5 days with NVP-BEZ235 45 mg/kg PO (bottom row). All mice were ENU-treated *Tsc2*^+- ^mice of age 24 weeks. Columns are: pS6(S235/236); S6 (total); pAKT(S473); AKT (total). Adjacent sections from each tumor are shown. All images are taken at 100×. Click here for file
