# Supplementary material for: Equivalent benefit of mTORC1 blockade and combined PI3K-mTOR blockade in a mouse model of tuberous sclerosis
Source: Mol Cancer. 2009 Jun 15;8:38. doi: 10.1186/1476-4598-8-38 (PMC2702302; doi:10.1186/1476-4598-8-38)
Supplement: Additional file 1 — Effects of ENU on kidney tumor development in Tsc2+- mice. A dot plot graph is shown of the gross kidney tumor scores in Tsc2+- mice at 6 months of age. E13, E19, P9, P21 indicates different ages of treatment with a single dose of ENU at 60 mg/kg IP. Each dot represents a single mouse. The differences among the 5 groups are statistically significant at p = 0.02; for the four ENU treatment groups, p = 0.035; for the three ENU treatment groups of E13, E19, and P9, p is not significant (0.79); all done by the Kruskall-Wallis test. [file 1476-4598-8-38-S1.pdf]

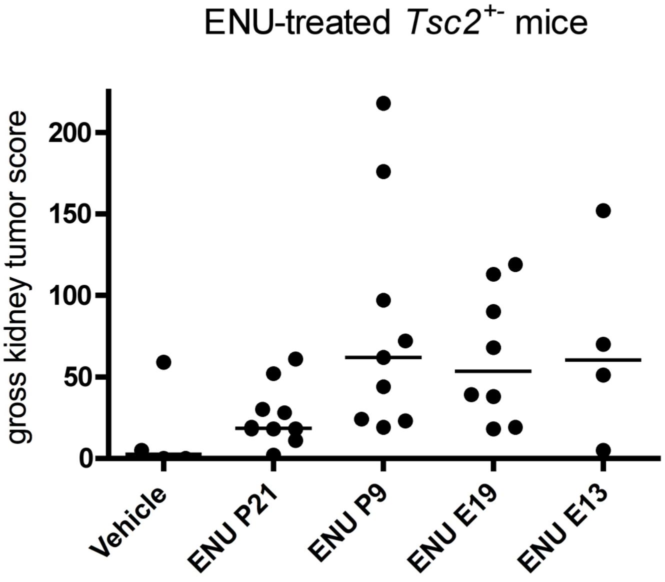

Supplemental Figure 1
Pollizzi et al.
