# Supplementary figures and images for: Equivalent benefit of mTORC1 blockade and combined PI3K-mTOR blockade in a mouse model of tuberous sclerosis
Source: Mol Cancer. 2009 Jun 15;8:38. doi: 10.1186/1476-4598-8-38 (PMC2702302; doi:10.1186/1476-4598-8-38)

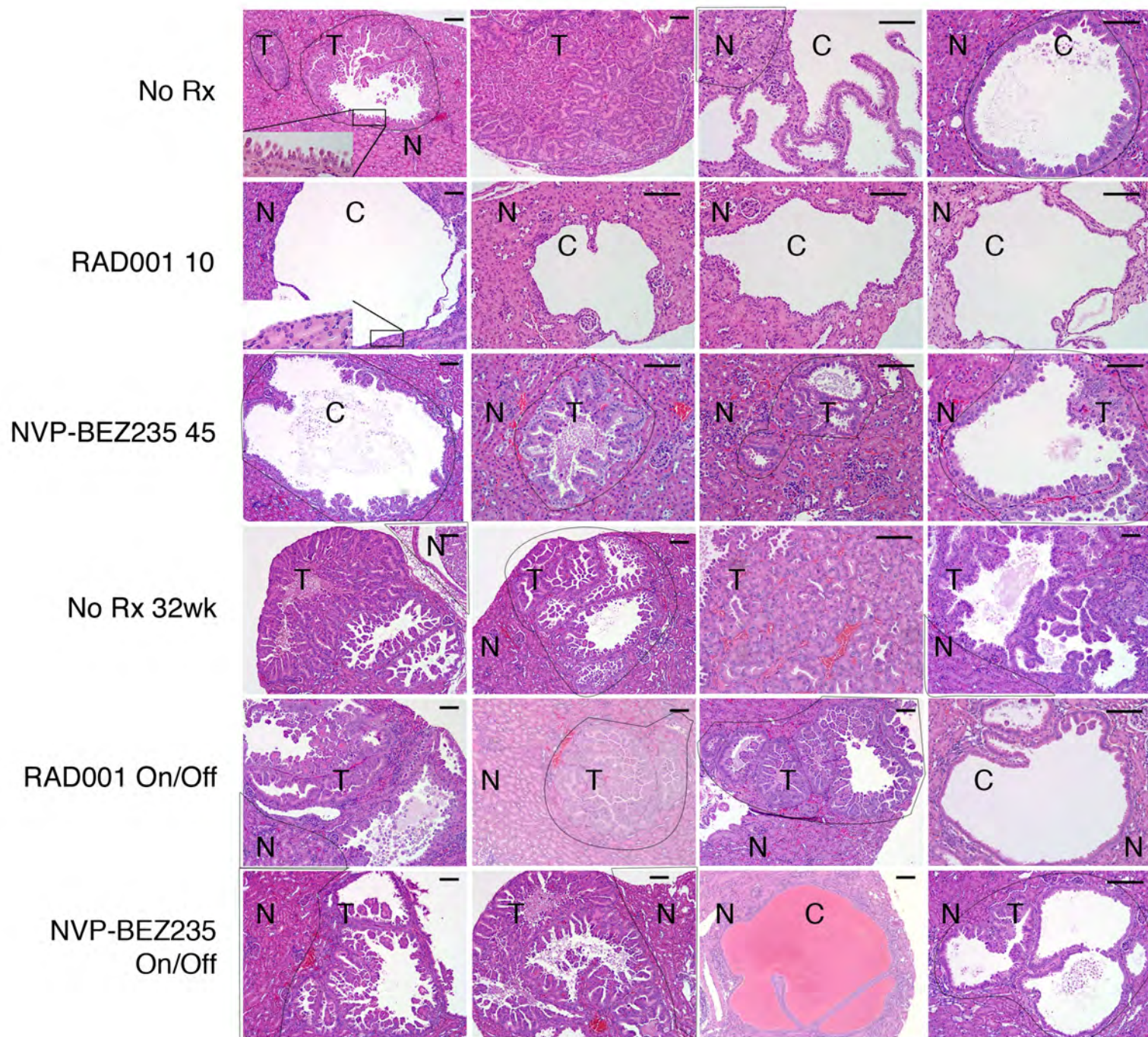

Supplemental Figure 2

Supplement: Additional file 2 — Morphology of RAD001- and NVP-BEZ235-treated Tsc2+- kidney lesions. Multiple tumor H&E stained images are shown from ENU-treated Tsc2+- kidneys. All scale bars are 100 microns. Each image is taken from a different mouse. Light black lines delineate tumor (T) or cyst (C) vs. normal kidney (N). Row A- Mice of age 24 weeks; inset shows hyperplastic columnar-type epithelium lining the cyst; Row B- Mice of age 24 weeks that were treated with RAD001 5 days per week, from age 20–24 weeks; inset shows flat epithelium lining the cyst: Row C- Mice of age 24 weeks that were treated with NVP-BEZ235 45 mg/kg daily from age 20–24 weeks; Row D- Mice of age 32 weeks; Row E- Mice of age 32 weeks that were treated with RAD001 5 days per week, from age 20–24 weeks; Row F- Mice of age 32 weeks that were treated with NVP-BEZ235 45 mg/kg daily from age 20–24 weeks. [file 1476-4598-8-38-S2.pdf]

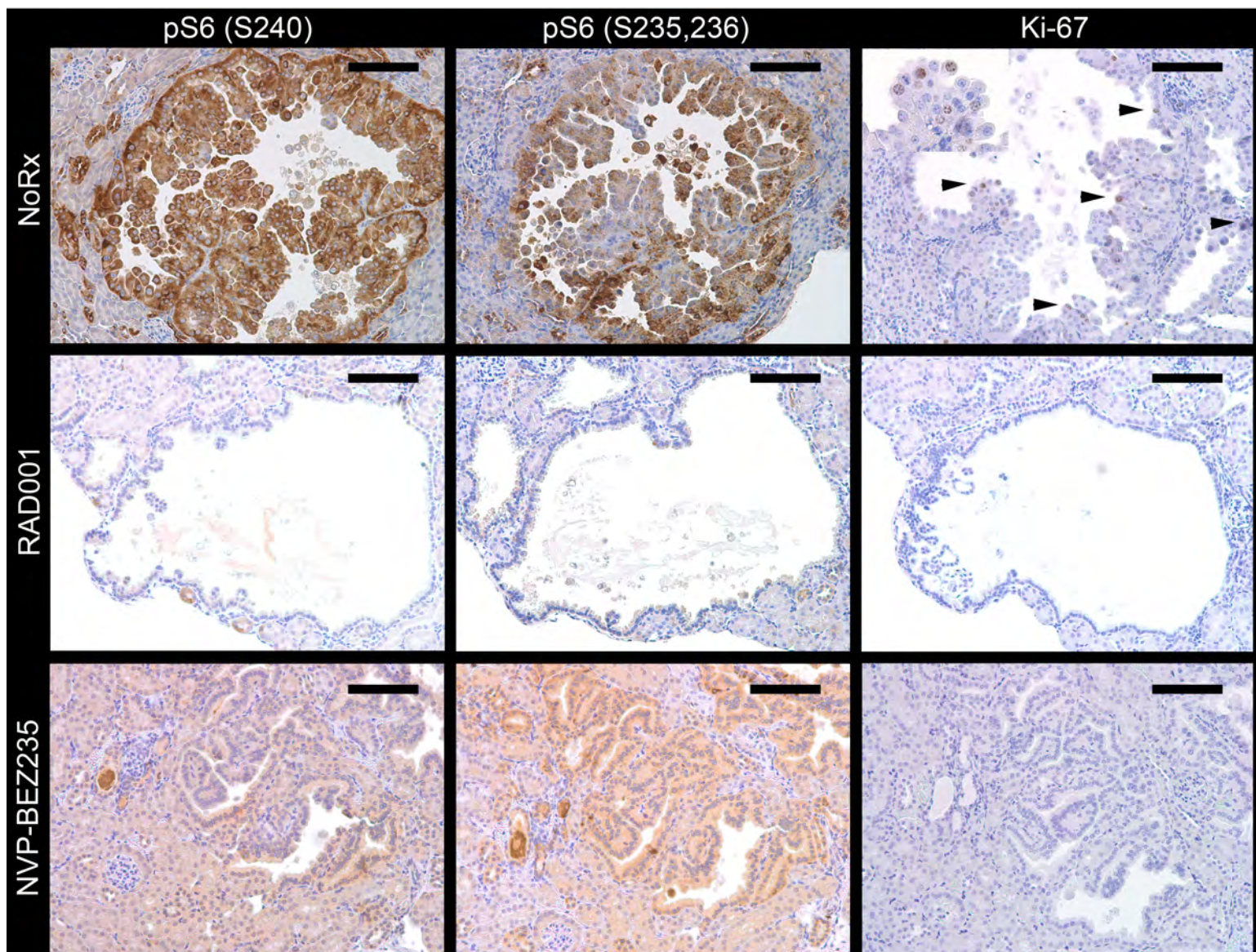

Supplemental Figure 3

Supplement: Additional file 3 — Signaling pathway and growth analysis by IHC in Tsc2+- mice. A single representative image is shown for untreated mice (top row), mice treated for 5 days with RAD001 10 mg/kg PO (middle row), and mice treated for 5 days with NVP-BEZ235 45 mg/kg PO (bottom row). All mice were ENU-treated Tsc2+- mice of age 24 weeks. Columns are: pS6(S240), pS6(S235/236), Ki-67. Adjacent sections from each tumor are shown. All images are taken at 100×. Note that the third row has a background pink/orange shade, which is present in all cells on this section, and distinct from the dark brown stain seen in the top row. [file 1476-4598-8-38-S3.pdf]

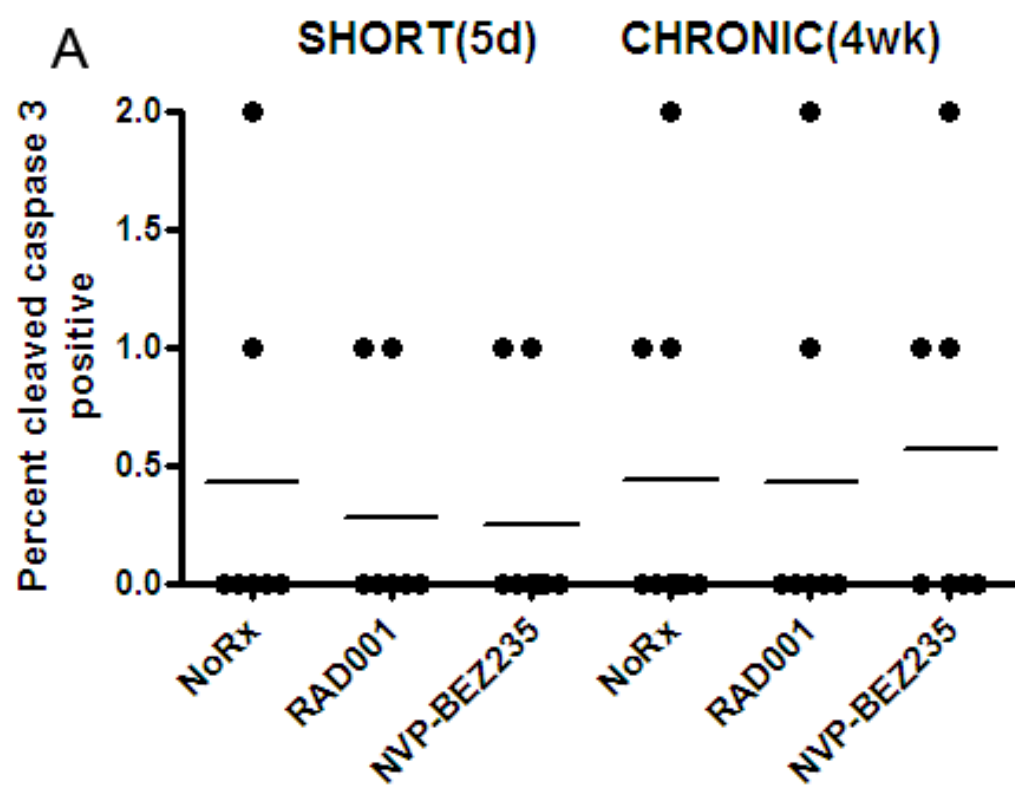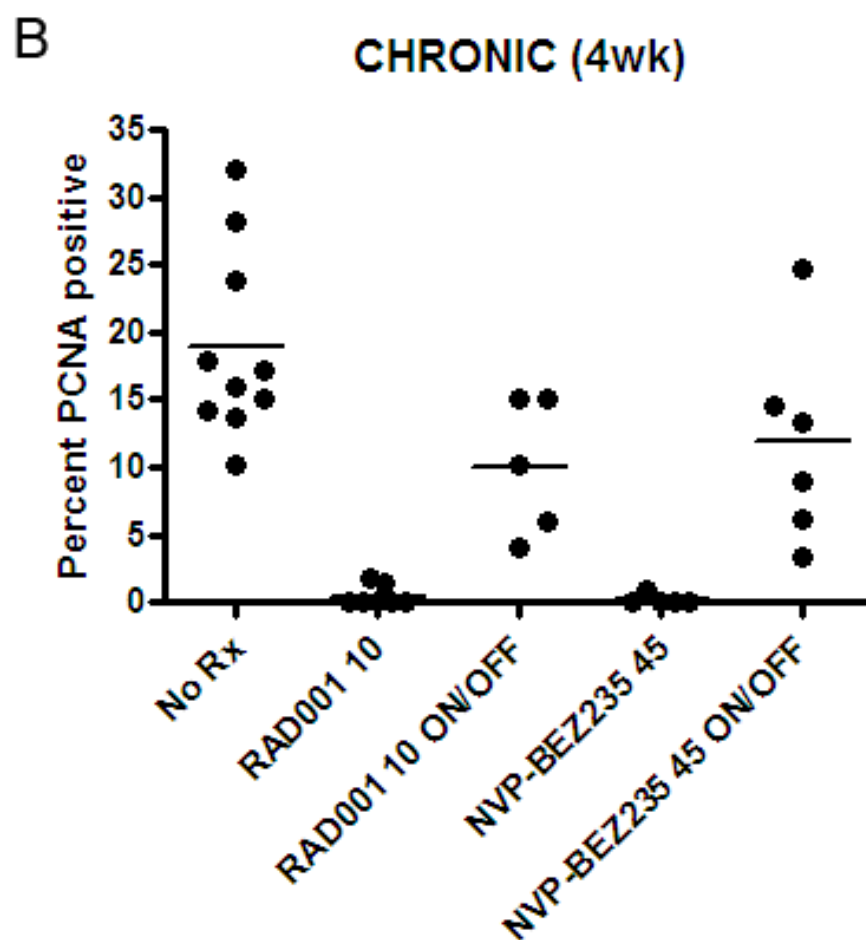

Supplement: Additional file 4 — Apoptosis and proliferation in Tsc2+- mice. A. The percent cleaved caspase 3 positive cells are shown for Tsc2+- mice, treated for either 5 days ('SHORT') or for 4 weeks ('CHRONIC'). Each dot represents a different renal cystadenoma for which the percent positive cells was determined by a blinded observer (IM). B. The percent PCNA positive cells are shown for Tsc2+- mice, treated with either RAD001 or NVP-BEZ235, or treated with these drugs and then taken off drug for 8 weeks ('ON/OFF'). Each dot represents a different renal cystadenoma. [file 1476-4598-8-38-S4.pdf]

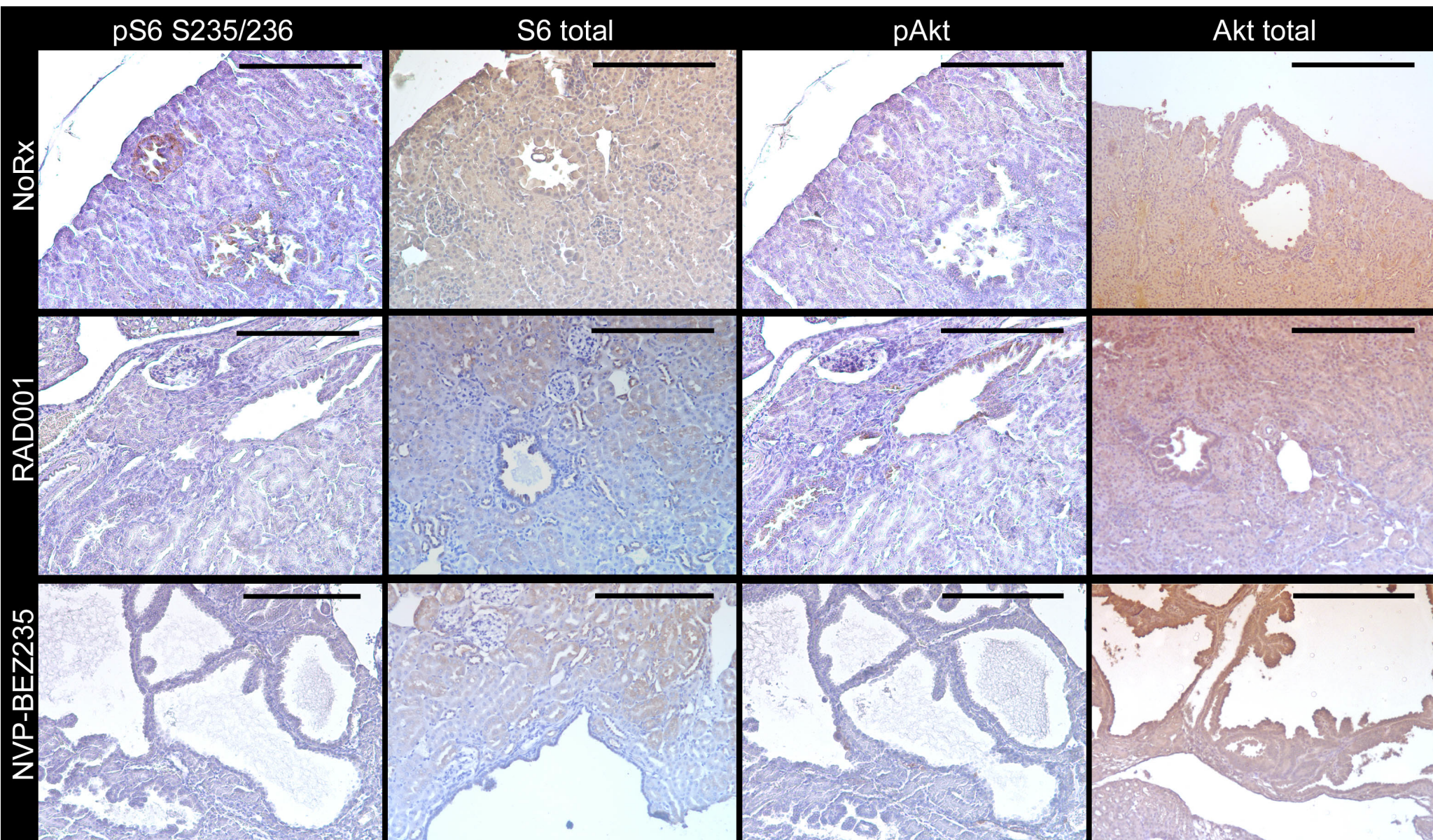

Supplement: Additional file 5 — Signaling pathway analysis by IHC in Tsc2+-mice. A single representative image is shown for untreated mice (top row), mice treated for 5 days with RAD001 10 mg/kg PO (middle row), and mice treated for 5 days with NVP-BEZ235 45 mg/kg PO (bottom row). All mice were ENU-treated Tsc2+- mice of age 24 weeks. Columns are: pS6(S235/236); S6 (total); pAKT(S473); AKT (total). Adjacent sections from each tumor are shown. All images are taken at 100×. [file 1476-4598-8-38-S5.pdf]
